# Ceria-Containing Hybrid Multilayered Microcapsules for Enhanced Cellular Internalisation with High Radioprotection Efficiency

**DOI:** 10.3390/molecules25132957

**Published:** 2020-06-27

**Authors:** N. R. Popova, A. L. Popov, A. M. Ermakov, V. V. Reukov, V. K. Ivanov

**Affiliations:** 1 Institute of Theoretical and Experimental Biophysics, Russian Academy of Sciences, Pushchino, Moscow Region 142290, Russia; nellipopovaran@gmail.com (N.R.P.); antonpopovleonid@gmail.com (A.L.P.); ao_ermakovy@rambler.ru (A.M.E.); 2 University of Georgia, 315 Dawson Hall, Athens, GA 30602, USA; reukov@gmail.com; 3 Kurnakov Institute of General and Inorganic Chemistry, Russian Academy of Sciences, Moscow 119991, Russia

**Keywords:** polyelectrolyte microcapsules, intracellular delivery, cerium oxide nanoparticles, nanoceria, radioprotection, oxidative stress

## Abstract

Cerium oxide nanoparticles (nanoceria) are believed to be the most versatile nanozyme, showing great promise for biomedical applications. At the same time, the controlled intracellular delivery of nanoceria remains an unresolved problem. Here, we have demonstrated the radioprotective effect of polyelectrolyte microcapsules modified with cerium oxide nanoparticles, which provide controlled loading and intracellular release. The optimal (both safe and uptake efficient) concentrations of ceria-containing microcapsules for human mesenchymal stem cells range from 1:10 to 1:20 cell-to-capsules ratio. We have revealed the molecular mechanisms of nanoceria radioprotective action on mesenchymal stem cells by assessing the level of intracellular reactive oxygen species (ROS), as well as by a detailed 96-genes expression analysis, featuring genes responsible for oxidative stress, mitochondrial metabolism, apoptosis, inflammation etc. Hybrid ceria-containing microcapsules have been shown to provide an indirect genoprotective effect, reducing the number of cytogenetic damages in irradiated cells. These findings give new insight into cerium oxide nanoparticles’ protective action for living beings against ionising radiation.

## 1. Introduction

Unique redox activity and low toxicity make nanoceria highly promising as a material for biomedical applications [1,2,3,4]. It has previously been shown that cerium oxide nanoparticles are capable of mimicking the activity of endogenous antioxidants, such as catalase and superoxide dismutase (SOD) [5,6]. The enzyme-like activity of nanoceria makes it possible to inactivate a wide range of free radicals and reactive oxygen species (ROS) [7]. Nanoceria reliably protect cells from UV radiation and various toxicants and are able to act as a mitogen stimulating stem cell proliferation [8,9,10]. The pH-dependent redox activity of nanoceria is used for the selective induction of apoptosis in cancer cells [11,12,13,14]. In particular, it has been shown that nanoceria modulate intracellular signalling pathways, causing selective death of fibrosarcoma cancer cells [15]. Dextran-stabilised cerium oxide nanoparticles have been used to prevent tumour invasion in tumour-stroma interaction [16]. Nanoceria can also act as a radiosensitiser inducing selective death of cancer cells upon exposure to radiation [17]. Cerium oxide nanoparticles modified with neogambogic acid potentiated the toxic effects of radiation in MCF-7 breast cancer cells, leading to a higher rate of cell death and inducing the activation of autophagy and cell cycle arrest in the G2/M phase. Nanoceria can also act as a radioprotector for normal tissue and cells. For example, we have previously shown that citrate-stabilised nanoceria have a complex mechanism of radioprotective action in vitro and in vivo [18,19]. The protective effect of citrate-stabilised nanoceria is due to their antioxidant activity through inactivation of ROS (hydroxyl radical and hydrogen peroxide) and a decrease in the level of cytogenetic damage in the bone marrow of irradiated animals. We have also shown that nanoceria activate the antioxidant defence system of animals by modulating the gene expression involved in the cellular response to radiation-induced oxidative stress.

Despite the significant therapeutic potential of nanoceria, there are still some unresolved problems hindering their widespread use in biomedical applications. Cerium oxide nanoparticles are hardly detected by commonly used luminescent methods, which inhibits the possibility of analysing their intracellular localisation, cellular processing and clearance. Thus, the design of a nanoceria intracellular delivery system capable of providing controlled delivery and dosage with the possibility of their bio-visualisation is a highly urgent task.

One possible effective intracellular delivery system may be designed using multilayer polyelectrolyte microcapsules. Polyelectrolyte capsules have shown enormous potential for being used in biomedical applications for cellular internalisation, tissue engineering or drug delivery [20,21,22,23,24,25]. Effective delivery of biologically active substances, low molecular weight compounds, RNA, DNA, and proteins using microcapsules have previously been reported [26,27,28,29,30]. To date, the physical and chemical characteristics of microcapsules, as well as the molecular mechanisms of their formation, have been well studied. Furthermore, various schemes for encapsulating and releasing biologically active substances from microcapsules have been proposed [31,32,33,34,35]. For example, stimuli-responsive microcapsules can release biologically active substances upon an external stimulus, e.g., upon a changing pH or an ionic microenvironment [36,37,38]. Multilayer microcapsules are effectively taken up by various cell types, which expands the technical capabilities of their practical use.

Here, we report on a new type of biodegradable microcapsule loaded with cerium oxide nanoparticles which are non-toxic for human mesenchymal stem cells and provide controlled loading and intracellular release. A comprehensive analysis of their cytotoxicity, biocompatibility, and cellular uptake using human mesenchymal stem cells was carried out. Nanoceria-loaded microcapsules ensured the efficient delivery of nanoparticles into the cell and provided efficient radioprotection from X-ray radiation. Results obtained give new insight into nanoceria radioprotective action for living beings against radiation.

## 2. Results and Discussion

**Figure 1:**
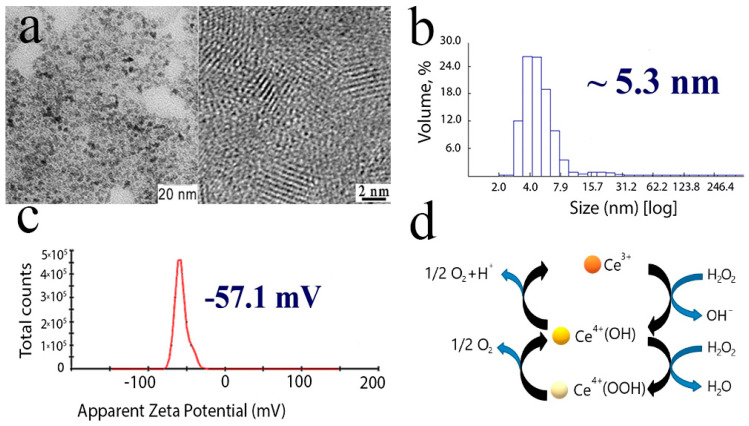
Cerium oxide nanoparticles: TEM and high-resolution TEM images (**a**), particle size distribution (**b**), zeta-potential (**c**), schematic representation of catalase-like activity of citrate-stabilised CeO_2_ nanoparticles (**d**).

Citrate-stabilised cerium oxide nanoparticles were 2–2.5 nm in size (Figure 1a), with a negative zeta potential (Figure 1c) and a hydrodynamic radius of about 5–7 nm when dispersed in water (Figure 1b). Citrate-stabilised cerium oxide nanoparticles demonstrated high enzyme-like antioxidant activity, as we have shown previously [39,40]. The scheme of antioxidant activity of nanoceria is shown in Figure 1d.

**Figure 2:**
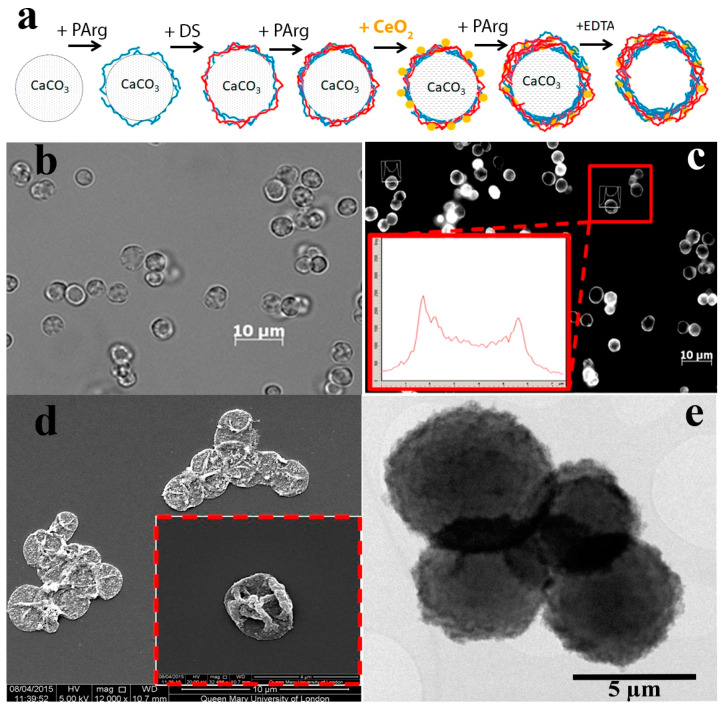
The scheme of biodegradable nanoceria-loaded polyelectrolyte microcapsules synthesis (**a**), laser confocal scanning microscopy in a bright field mode (**b**), laser scanning confocal microscopy in a fluorescence mode (inset - microcapsule fluorescence profile) (**c**), SEM (**d**), TEM (**e**) of nanoceria-loaded polyelectrolyte microcapsules.

The layer-by-layer (LbL) method was used to synthesise microcapsules, which were then modified with citrate-stabilised cerium oxide nanoparticles by replacing the negatively charged layer of polyelectrolyte (Figure 2a). The high negative zeta potential of cerium oxide nanoparticles made it possible to integrate them into the polyelectrolyte matrix. For the synthesis of microcapsules, biodegradable (40 kDa dextran sulphate and 15–70 kDa poly-L-arginine) polyelectrolytes were used. The high surface area of ultra-small cerium oxide nanoparticles determines their active interaction with various ions and biomolecules, which, ultimately, can lead to the loss of the enzyme-like properties of the nanoparticles. However, the integration of nanoceria into the polyelectrolyte microcapsule can protect the encapsulated substance from negative external factors, as we have shown earlier [41]. The integration of nanoceria into the middle layer of the microcapsule maintains the possibility of access of various forms of ROS and free radicals to the surface of nanoparticles, due to the porous structure of the polyelectrolyte matrix. Thus, the optimal location of nanoceria integration for efficient intracellular delivery is in the middle layer of the polyelectrolyte microcapsule.

The synthesised microcapsules had a round shape and a size of about 3–4 μm (Figure 2b). It is worth noting that ceria sol used for the preparation of microcapsules possessed only weak luminescence; however, integration of cerium oxide nanoparticles into the polyelectrolyte matrix led to the appearance of strong fluorescence (Figure 2c), which allowed microcapsules to be visualised while working with cell cultures. According to existing reports, cerium oxide nanoparticles can acquire luminescent properties due to the formation of electronic defects in the crystal structure (the presence of Ce^3+^ ions) [42,43,44]. Basically, the cerium ions in CeO_2_ are in the +4 valence state. Ce^4+^ has a 4f^0^electronic configuration, and does not exhibit luminescent properties, unlike Ce^3+^, which has a 4f^1^ configuration, and has an intense 5d-4f luminescence band, which is located, depending on the crystal matrix, in the near UV or visible region. From the point of view of spectroscopy, CeO_2_ can be attributed to the class of compounds whose optical properties are determined by electronic transitions in charge transfer complexes from the ligand to the metal ion. The analysis of the morphological characteristics of nanoceria-loaded microcapsules by scanning electron microscopy revealed a characteristic morphology (surface granulation) of the microcapsules, which confirmed the integration of cerium oxide nanoparticles into the polyelectrolyte matrix (Figure 2d). The tendency of microcapsules to aggregate should also be noted. The characteristic deformation of microcapsules is associated with the technique of sample preparation for SEM analysis, which includes the drying stage. The morphological characteristics of nanoceria-loaded microcapsules were further analysed by transmission electron microscopy (Figure 2e). It was shown that cerium oxide nanoparticles were readily incorporated into the polyelectrolyte matrix, and this was clearly seen in the formation of electron-dense areas on the surface of the microcapsule. EDX analysis confirmed the effective loading of nanoceria into the middle layer of microcapsules.

**Figure 3:**
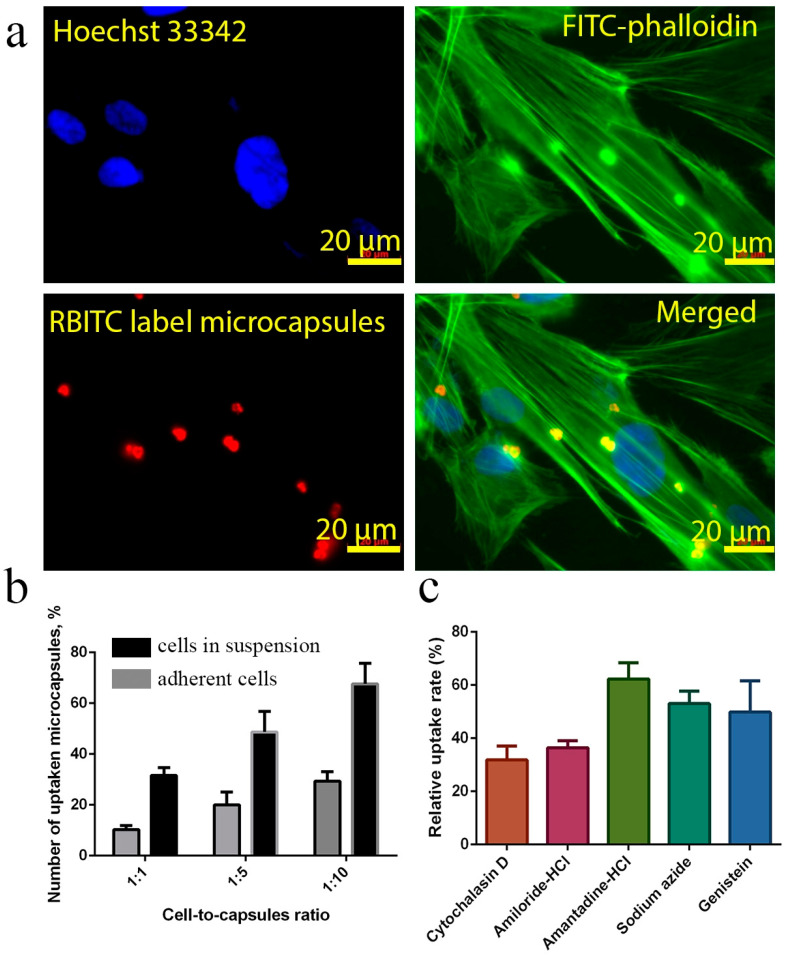
Cellular uptake of nanoceria-loaded polyelectrolyte microcapsules by human mesenchymal stem cells (hMSC) after 24 h of incubation (**a**). Rhodamine B isothiocyanate (RBITC)-labelled microcapsules (red) were used. The actin cytoskeleton and nucleus were stained with fluorescein isothiocyanate (FITC) conjugated phalloidin (green) and Hoechst 33,342 (blue), respectively. The scale bar is 20 μm. The uptake efficiency of nanoceria-loaded polyelectrolyte microcapsules: addition of microcapsules to the cell suspension or to the adherent cells (**b**). Molecular mechanisms of microcapsules endocytosis (**c**). The hMSC were cultured with pretreatment by different pharmacological inhibitors: amantadine-HCl (1 mM), genistein (200 mM), amiloride-HCl (1 mM), NaN_3_ (40 mM) and cytochalasin D (CytD, 10 µg/mL) for 1 h. Cell counts associated with microcapsules were taken from ten micrographs.

Next, we analysed the endocytosis pathways of nanoceria-loaded microcapsules. It has previously been shown that microcapsules can be taken up by various cell types [45]. The uptake efficiency of microcapsules may depend on their size [46], charge of the outer layer [47], the type of polyelectrolytes used [48], the elasticity or deformability of the microcapsule shell [49] or their shape [50]. Here, human mesenchymal stem cells were used to analyse the endocytosis of nanoceria-loaded microcapsules using fluorescence microscopy (Figure 3a). We used two different experimental designs for evaluating the endocytosis process. In the first case, microcapsules were introduced directly into the cell suspension before seeding, and in the second case, they were added to adherent cells. It was shown that the addition of microcapsules into the suspension of cells significantly increased their percentage uptake by the cells (Figure 3b). In a ratio of 1:10, a 2.5-fold increase in uptake efficiency was shown, compared with addition to attached cells. Apparently, this was due to the actin cytoskeleton of the cell, which is less labile for restructuring in the process of endocytosis of the microcapsule when the cell is in an attached state. We also performed additional studies using the Z-stack option of Laser Confocal Scanning Microscopy (LCSM), which confirmed that the microcapsules were located directly in the cell cytoplasm and not on the cell surface (Figure S2). The uptake mechanism of nanoceria-loaded microcapsules was further studied using pharmacological inhibitors (Figure 3c). The following inhibitors were used: amantadine (1 mM, an inhibitor of clathrin-mediated endocytosis), genistein (200 mM, caveolin-mediated endocytosis), amiloride (1 mM, an inhibitor of macropinocytosis), NaN_3_(40 mM, an inhibitor of the energy-dependent processes), andcytochalasin D (10 μg/mL, actin cytoskeleton inhibitor). There are several possible ways to internalise exogenous particles in a cell, including clathrin-mediated endocytosis, caveolin-mediated endocytosis, clathrin-caveolin-independent endocytosis, and macropinocytosis. Macropinocytosis may be blocked by amiloride due to inhibition of Na^+^/H^+^ channels; clathrin-mediated endocytosis can be inhibited by amantadine by preventing the formation of vacuolar vesicles coated with clathrin; caveolin-mediated endocytosis is inhibited by genistein by blocking the Src-tyrosine kinase family; the cytoskeleton can be destroyed using cytochalasin D, which has a strong effect on the transporting of particles into the cell. The uptake of nanoceria-loaded microcapsules was completely blocked by cytochalasin and amiloride, and, to a lesser degree, blocked by sodium azide. These results indicate that the uptake of microcapsules is a process that is energydependent. The use of amantadine did not affect the uptake of nanoceria-loaded microcapsules, indicating that the clathrin-mediated absorption method had almost no effect on the internalisation of microcapsules. Cytochalasin D suppressed uptake efficiency, which confirms the active participation of the cytoskeleton in the process of microcapsule endocytosis. Thus, the main means of microcapsule penetration was energy-dependent macropinocytosis. Meanwhile, it is worth noting that the analysis of the efficiency of microcapsule absorption was complicated by their aggregation at the cell surface and their adsorption on the cell membrane without internalisation.

**Figure 4:**
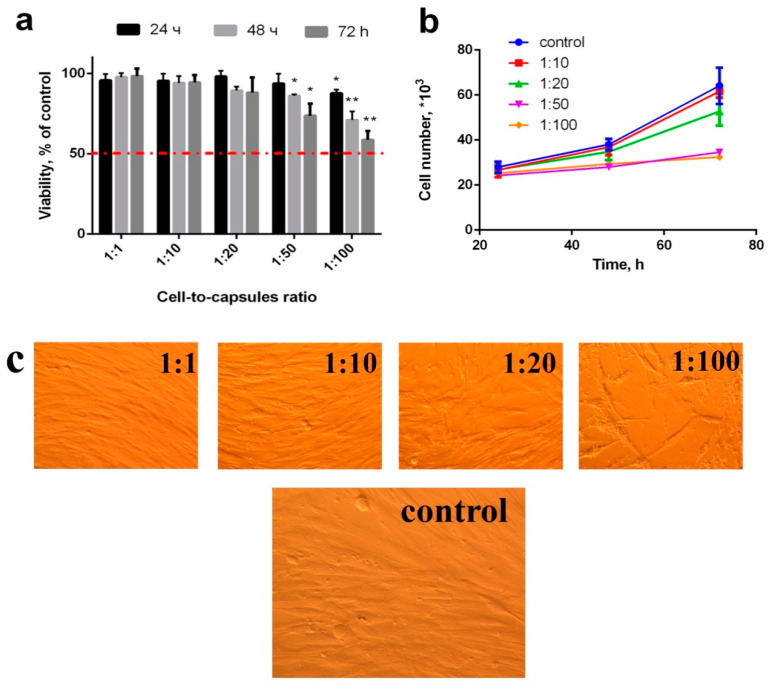
Viability (via MTT assay) (**a**), proliferation (**b**) and morphology appearance (**c**) of hMSC after incubation with nanoceria-loaded polyelectrolyte microcapsules. Significant differences compared with the control using the *t*-test with Welch’s correction, * *p* < 0.05; ** *p* < 0.0001. Control: without the addition of microcapsules.

To analyse the cytotoxicity of the microcapsules, they were added to the adherent cell culture 6 h after attachment (Figure 4). It was shown that high concentrations of nanoceria-loaded microcapsules (more than 50 per cell) inhibited their proliferation and metabolic activity without causing cell death (Figure 4b). At the same time, concentrations from 1 to 20 microcapsules per cell did not cause any changes in proliferative activity of all types of cell cultures during 24, 48, and 72 h of cultivation. Using MTT assay, it was shown that concentrations of 50 and 100 microcapsules per cell did not cause a decrease in the level of dehydrogenase activity (Figure 4a). No morphological changes were observed in human mesenchymal stem cell culture (Figure 4c). Note that only very high concentrations of microcapsules are toxic to mesenchymal stem cells, which is in line with results reported by other authors [51,52,53].

**Figure 5:**
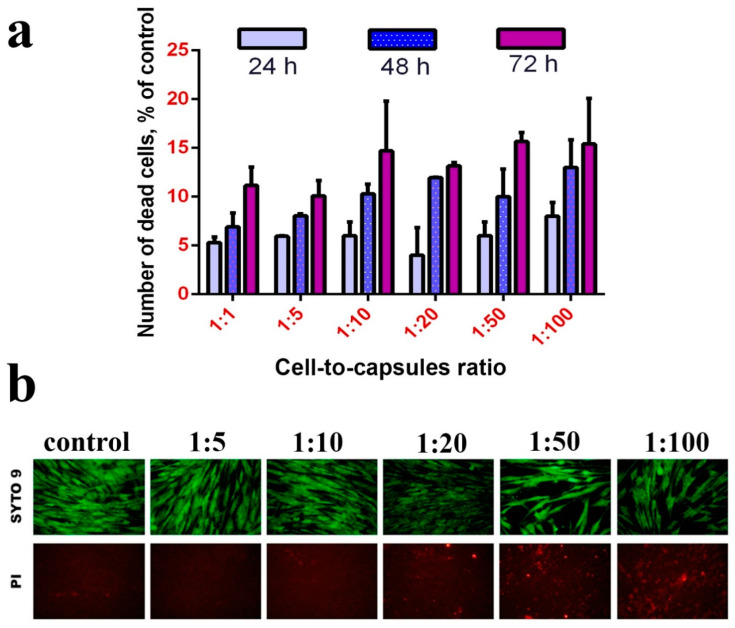
LIVE/DEAD assay of hMSC after incubation (24, 48 and 72 h) with nanoceria-loaded polyelectrolyte microcapsules (**a**). Micrographs of hMSC after incubation (24 h) with nanoceria-loaded polyelectrolyte microcapsules (**b**). Control: without the addition of microcapsules.

The analysis of cell culture viability using fluorescence microscopy (LIVE/DEAD assay) did not reveal a significant increase in the quantity of dead (positively stained with propidium iodide) cells (Figure 5). It is worth noting that the microcapsules themselves absorb propidium iodide and thus become stained. At large magnifications, stained microcapsules can easily be discriminated from the nuclei of dead cells allowing for the quantitative analysis of the latter (Figure S7). Upon treatment with a high concentration of microcapsules, the cells slightly increase in size, but do not lose their flatness and fusiform shape.

No significant differences were revealed in the quantity of cells with fragmented chromatin upon incubation with the composite microcapsules (at concentrations from 1 to 20 microcapsules per cell), which proves the absence of the genotoxic effect of these microcapsules.

**Figure 6:**
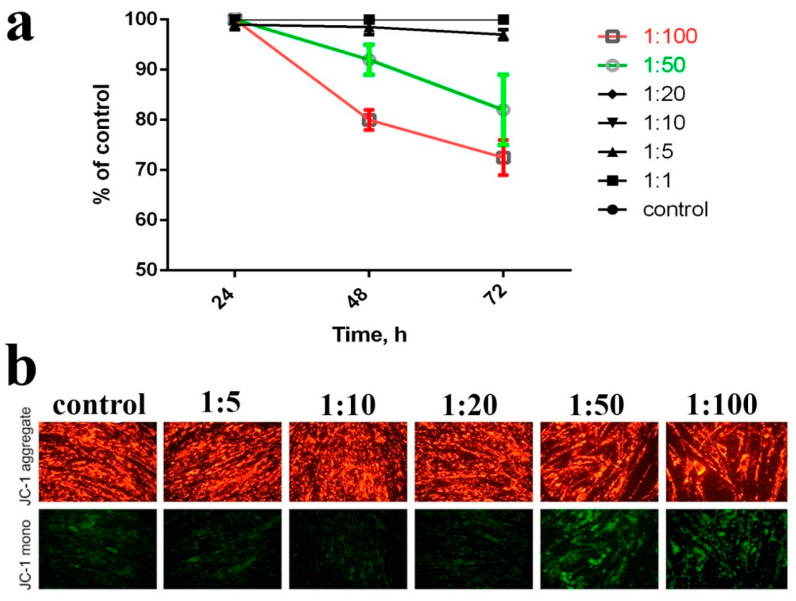
The effect of nanoceria-loaded polyelectrolyte microcapsules on the mitochondrial membrane potential (MMP) in hMSC after 24, 48, and 72 h of incubation (**a**). Micrographs of hMSC with JC-1 dye after incubation (24 h)with nanoceria-loaded polyelectrolyte microcapsules (**b**). Cells were plated in 96-well plates and left overnight. Then, nanoceria-loaded polyelectrolyte microcapsules were added, and after 24, 48, and 72 h, cells were stained with JC-1 dye. Control: without the addition of microcapsules.

Oxidative stress is the major mechanism of the cytotoxic action of the metal-based nano- and biomaterials. Nanoparticles can directly generate ROS on their surface, disrupting cellular metabolism and signalling, as well as indirectly inducing, ROS generation in mitochondria, promoting oxidative stress in the cell. Since nanoceria are a redox-active nanomaterial, we decided to study the effect of nanoceria-loaded microcapsules on the mitochondrial membrane potential (MMP). The study of MMP after incubation with nanoceria-loaded microcapsules in the concentration range from 1 to 20 microcapsules per cell did not reveal any significant differences with the untreated control (Figure 6). In turn, higher concentrations of microcapsules (50 and 100 microcapsules per cell) caused a decrease in MMP to 60–70% of the control values. Based on these data, nanoceria-loaded microcapsules can be judged to be biocompatible. MMP is an indicator of cell metabolic activity, and the decrease of MMP may indicate certain abnormalities in the mitochondria functioning, the development of mitochondria-mediated apoptosis or the generation of excess ROS due to the uncoupling of oxidative phosphorylation.

**Figure 7:**
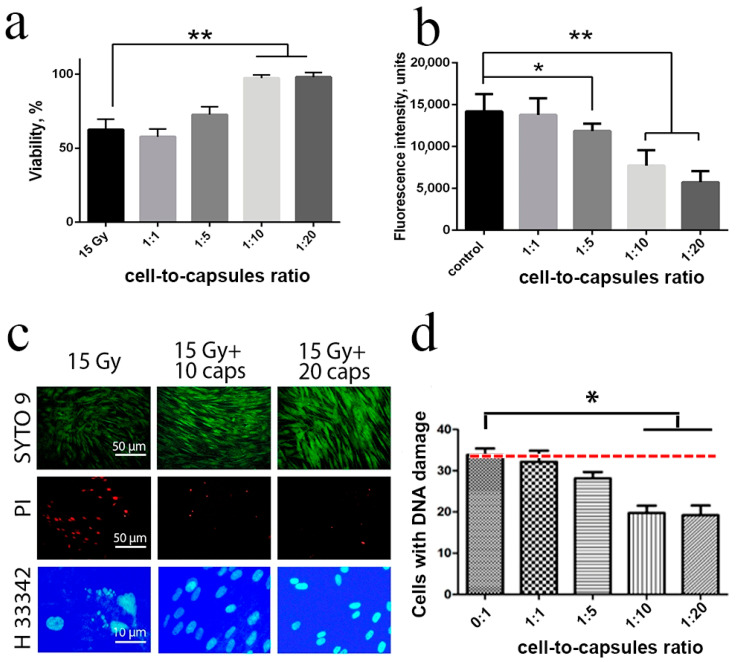
Viability (via MTT assay) of human mesenchymal stem cells pre-treated with nanoceria-loaded polyelectrolyte microcapsules 48 h after exposure to X-rays at a dose of 15 Gy (**a**). Reactive oxygen species (ROS) level determination by staining with dichlorofluorescein (**b**). Live/Dead assay and micronuclei analysis after X-ray irradiation of cells initially treated with nanoceria-loaded polyelectrolyte microcapsules 48 h after exposure (**c**). Quantitative analysis of micronuclei in hMSC treated with nanoceria-loaded microcapsules after X-ray irradiation (15 Gy) (**d**). *—Significant differences as estimated by *t*-test with Welch’s correction, * *p* < 0.05, ** *p* < 0.0001, Control—X-ray irradiation (15 Gy) of the cells without nanoceria-loaded polyelectrolyte microcapsules. Cells were treated with nanoceria-loaded polyelectrolyte microcapsules 24 h before irradiation.

In addition, the radioprotective properties of nanoceria-loaded microcapsules were investigated. First, we experimentally determined the optimal cell density, exposure/cultivation time and other parameters of X-ray exposure testing of human mesenchymal stem cells (Figure S1, Figure 3, Figure 4, and Figure 5). It was shown that the optimal slot for analysing cell viability was 72 h after irradiation, with a cell density of 20,000 per cm^2^ (Figure S3). In living cells, nanoceria-loaded microcapsules begin to degrade due to the action of endogenous enzymes, and to release cerium oxide nanoparticles into the cytoplasm. Evenly distributed in the cytoplasm, cerium oxide nanoparticles efficiently protect the cells from the negative effects of ionising radiation (Figure 7a). We analysed the level of intracellular ROS after irradiation, considering water radiolysis to be the main cause of the ionising radiation damages, and nanoceria to be an efficient ROS scavenger (Figure 7b). It was found that nanoceria-loaded microcapsules (10 and 20 capsules per cell) significantly reduced the intracellular ROS level after 15 Gy X-ray irradiation (*p* ≤ 0.005). At the same time, lower concentrations (1 and 5 capsules per cell) did not provide an adequate protective effect. We confirmed this radioprotective efficiency by analysing the number of dead cells and cells with fragmented DNA (Figure 7c). Nuclei fragmentation after X-ray exposure (15 Gy) is a clear indicator of apoptosis or cell death. Preincubation of the cells with nanoceria-loaded microcapsules prevents nuclei fragmentation, as confirmed by microphotographs (Figure 7c). Considering the fact that nanoparticles cannot penetrate the cell nucleus, we can propose that nanoceria exhibit an indirect genoprotective effect through a decrease of ROS level or by affecting the DNA repair process. It was shown that pre-incubation of the cells with nanoceria-loaded microcapsules (10 and 20 capsules per cell) increased survival rate after irradiation up to control values, which indicated the highest degree of radioprotection. Given the size of the cell and its ability to take up a limited number of microcapsules, it is worth noting that such a quantity of microcapsules (10 or 20 pieces per cell) was excessive. However, the radioprotective effect of a lower concentration of microcapsules (5 pieces per cell) was insufficient, which indirectly confirmed the different efficiency of microcapsule endocytosis. It was also shown that cellular uptake of microcapsules decreased the number of cells with damaged DNA, in a dose-dependent manner (Figure 7d).

Thus, we can state that the radioprotective action of nanoceria-loaded microcapsules was ensured by the decrease in intracellular ROS level, significantly reducing the number of dead cells, as well as reducing the proportion of cells with fragmented DNA.

**Figure 8:**
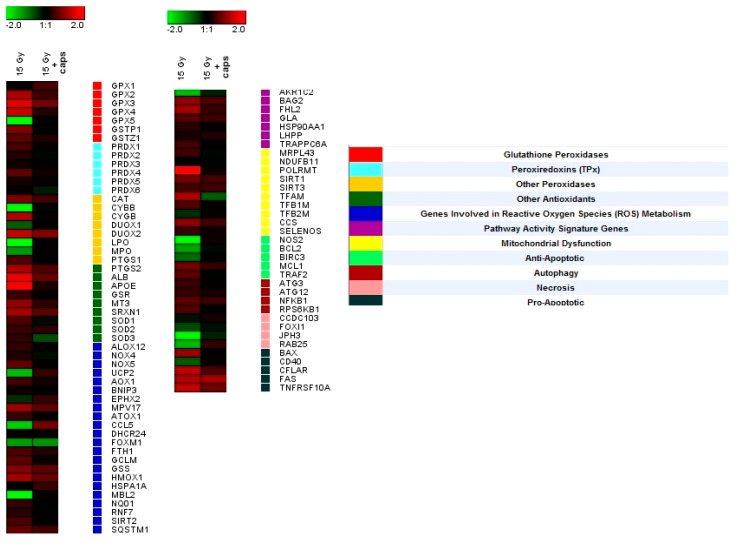
Heat map of gene expression in hMSC after 24 h of incubation with nanoceria-loaded polyelectrolyte microcapsules (10 capsules per cell). Nanoceria-loaded microcapsules were added to the adherent cells. The intensity scale of the standardised expression values ranges from –5 (green: low expression) to +5 (red: high expression), with a 1:1 intensity value (black) representing the control (cells not treated with microcapsules).

Gene expression analysis was performed to reveal the molecular mechanisms of nanoceria-loaded microcapsules’ radioprotective action (Figure 8). Using real-time PCR, expression levels were obtained of 96 genes involved in the cellular response to oxidative stress induced by X-ray irradiation. The analysis indicated that exposure to X-rays led to the activation of the gene groups responsible for apoptosis, necrosis, autophagy, signalling pathways involved in ROS metabolism, etc. (genes ALB, APOE, GPX4, CYGB, SIRT1, TNFRSF10A, FAS, BAX, and NOS). Upon pretreatment of the hMSC with nanoceria-loaded microcapsules (20 capsules per cell), the gene expression remained at the initial level, which indirectly confirmed cerium oxide nanoparticles’ ability to mimic the activity of endogenous antioxidants, such as superoxide dismutase (SOD) and catalase. However, the molecular mechanisms of the nanoceria-loaded microcapsules’ radioprotective action require further in-depth study, including analysis of the metabolic and signalling pathways after exposure to microcapsules and ionising radiation.

Successful encapsulation of nanoceria into polyelectrolyte microcapsules and their pronounced radioprotection efficiency will pave the way to novel multimodal radioprotective preparations by co-encapsulation of nanoceria with synergetic drugs [54,55,56]. The proposed microcapsule structure not only provides controlled intracellular delivery of nanoceria and preservation of its antioxidant activity, but also makes it possible to perform combined synergistic drug delivery. To date, polyelectrolyte microcapsules have not been used for radioprotection; therefore, our data significantly expand the scope of their biomedical application. Our work also further advances therapeutic biomaterials based on nanocrystalline cerium oxide. Recently, it has been shown that nanocrystalline cerium oxide is a very promising nanomaterial for radioprotection of normal tissues [56,57,58,59,60,61] while its practical applications are considerably hindered by the absence of robust delivery systems. The use of polyelectrolyte microcapsules provides the means for a precisely dosed delivery of cerium oxide nanoparticles into the cell with their subsequent controlled release, as well as providing a pronounced therapeutic effect, which is confirmed by our current results. The advantage of microcapsules is an accurate dosage of the loaded substance and controlled release into the cell, which is important when developing any therapeutic drugs, including radioprotective agents, since high concentrations of the vast majority of radioprotectors are toxic. Any further studies of the radioprotective properties of ceria-loaded microcapsules should take into account the safe concentrations established in this paper.

## 3. Materials and Methods

### 3.1. Materials

Dextran sulphate sodium salt (DS, molecular weight ≈ 10 kDa, #D4911) and poly-L-arginine hydrochloride (PArg, MW ≈ 70 kDa, #P3892), calcium chloride (CaCl_2_, #223506), sodium carbonate (Na_2_CO_3_, #S7795), ethylenediaminetetraacetic acid disodium salt dihydrate (Na_2_EDTA 2H_2_O, #E5134), amantadine-HCl (#A1260), genistein(#G6649), amiloride-HCl(#A7410), NaN_3_ (#199931), cytochalasin D (#C2618),and Hoechst 33,342 (#14533) were purchased from Sigma-Aldrich (St. Louis, Missouri, USA). All chemicals were used as received. Ultrapure water with a resistance greater than 18.2 MΩ cm^−1^ was used for all experiments. Syto 9 (#S34854) and propidium iodide (#BMS500PI), JC-1 (#T3168) and fluorescein isothiocyanate (FITC)-phalloidin (#F432) were purchased from Thermo Fisher Scientific (Waltham, MA, USA).

### 3.2. Synthesis and Preparation of CeO_2_Nanoparticles

Aqueous colloid solutions of citrate-stabilised cerium oxide nanoparticles were synthesised using a previously reported technique [62]. According to transmission electron microscopy data, the sol obtained consisted of slightly aggregated CeO_2_ nanoparticles (2.0–2.5 nm), which had a nearly spherical isotropic shape. Zeta potential was found to be negative (–57 mV). The average CeO_2_ hydrodynamic radius was found to be 5.3 nm.

### 3.3. Synthesis of CeO_2_ Composite LbL Microcapsules

Calcium carbonate templates were fabricated as described previously [63]. The process was initiated by rapid mixing of equal volumes of 0.33 M aqueous solutions of CaCl_2_ and Na_2_CO_3_ at room temperature. After intense agitation with a magnetic stirrer for 30 s, the precipitate was separated by sedimentation, and rinsed with water three times. The sedimented CaCO_3_ microparticles were centrifuged at 1000 rpm for 1 min and then redispersed in water. This procedure formed an aqueous suspension containing spherical CaCO_3_ microparticles with an average diameter ranging from 2 to 3 µm. Calcium carbonate microparticles were used as a template for fabrication of the nanocomposite shells. The first polyelectrolyte layer was made by adsorption of the positively charged PArg from 1 mg mL^−1^ solution in 0.15 M NaCl (15 min of incubation and shaking) on CaCO_3_ microparticles dispersed in this solution. The second layer was prepared by absorption of the negatively charged DS from 1 mg mL^−1^ solution in 0.15 M NaCl (15 min of incubation and shaking). The core/polyelectrolyte microparticles were washed three times with deionised water after each adsorption step. A colloidal solution of CeO_2_ nanoparticles (10^–5^ M) was used to make the fourth layer in the microcapsule. The calcium carbonate cores were further dissolved in 0.2 M ethylenediaminetetraacetic acid (EDTA·2H_2_O) for 30 min. Finally, the microcapsules were centrifuged and rinsed three times with EDTA, and then three times using water.

### 3.4. Zeta-Potential Measurements

The ζ-potential of nanoceria-loaded microcapsules was measured on a Zetasizer Nano-ZS analyser (Malvern). Each value of ζ-potential was obtained under ambient conditions by averaging three independent measurements of 100 sub-runs each.

### 3.5. Scanning Electron Microscopy (SEM) Studies

The morphology of the microcapsules was studied by SEM (FEI Inspect-F) at an accelerating voltage of 20 kV. The diluted microcapsule suspension was placed on a glass slide, air dried and coated with gold before observation. The microcapsule size was calculated using Image-Pro Plus Version 6.0 analysis software (Media Cybernetics Inc., Rockville, USA). The microcapsule diameter and distribution were expressed as mean ± SD by randomly averaging the diameter of at least 35 capsules per sample from the SEM data.

### 3.6. Energy Dispersive X-Ray Spectroscopy (EDX)

Elemental analysis was performed by means of an EDX Oxford Inca X-act detector attached to the SEM, operating at an accelerating voltage of 20 kV.

### 3.7. Transmission Electron Microscopy (TEM)

The morphology and structure of microcapsules, as well as the size distribution of CeO_2_ particles and shell thicknesses, were further studied using a JEOL 2010 transmission electron microscope with a LaB_6_ filament, operated at 200 kV. For TEM experiments, the diluted microcapsule suspension was dropped on a copper grid with a holey carbon film and left to dry for 5 min.

### 3.8. Confocal Laser Scanning Microscopy (CLSM)

CLSM images were captured with a Leica TS confocal scanning system (Leica, Mannheim, Germany) equipped with a 63×/1.4 oil immersion objective.

### 3.9. Cell Culture

Human mesenchymal stem cells (hMSC) were isolated from the third molar germ extracted for orthodontic indications from a healthy 16 year-old patient. The cells were extracted with Dulbecco’s Modified Eagle’s Medium (DMEM) (PanEko, Moscow, Russia) supplemented with 200 U/mL penicillin and 200 mg/mL streptomycin (Life Technologies, USA) and further treated with 0.25% trypsin + 0.02% EDTA (Life Technologies, Carlsbad, California, USA) for 30 min at 37 °C. The isolated cells were centrifuged for 2 min at 1500 rpm and resuspended to a single cell state in the culture medium consisting of DMEM/F12 (1:1; Life Technologies, Carlsbad, California, USA) with 10% fetal bovine serum (FBS). The obtained cell suspension was transferred into 25 cm^2^ cell flasks and cultured in DMEM (PanEko, Moscow, Russia) supplemented with 10% FBS (HyClone, New York, USA), 100 U/mL penicillin/streptomycin, 2 mM L-glutamine at 37 °C and 5% CO_2_. When the subconfluent cells state was achieved, the cultured cells were treated with 0.25% EDTA/trypsin solution and transferred to 75 cm^3^ vials in a ratio of 1:3. Cells were cultured in DMEM/F12 (PanEko, Moscow, Russia) supplemented with 10% FBS, 100 U/mL penicillin/streptomycin, and 2 mM L-glutamine. In this study, cells were used between passages 3 and 4.

### 3.10. MTT Assay

Determination of the activity of mitochondrial and cytoplasmic dehydrogenases of living cells was performed by an MTT assay based on the reduction of colourless tetrazolium salt (3-[4,5-dimethylthiazol-2-yl]-2,5-diphenyltetrazolium bromide). Cells were seeded in 96-well plates and cultured in an atmosphere containing 5% CO_2_ at 37 °C. Six hours after cell seeding, the medium was replaced with the medium containing the microcapsules in an amount of 1, 10, or 100 microcapsules per cell. Within 24, 48, and 72 h of the addition of the microcapsules, viability was determined.

### 3.11. Live–Dead Assay

Assessment of the viability of the cells cultured in the presence of nanoceria-loaded microcapsules was performed on a Carl Zeiss Axiovert 200 microscope. An L-7007 LIVE/DEAD BacLight Bacterial Viability Kit (Invitrogen, Carlsbad, California, USA) was used for the assay, which included a SYTO 9 fluorescent dye (absorption/emission ~420/580 nm) and a propidium iodide (PI) dye (absorption/emission ~488/640 nm). The dyes were added to the medium (1 μg/mL) and the plate was placed in a CO_2_ incubator for 15 min. Then cells were rinsed with phosphate-buffered saline and microphotographs were taken.

### 3.12. Cellular Uptake of CeO_2_ Composite Microcapsule

Two experimental approaches were used to evaluate nanoceria-loaded microcapsule cell internalisation. In the first experimental approach, nanoceria-loaded microcapsules had been added to hMSC, which were attached in Petri dishes. In the second approach, nanoceria-loaded microcapsules were directly put into the suspension of hMSC and then the mixture obtained was seeded. After 24 h of incubation, the cells were washed with Hanks’ Balanced Salt Solution (HBSS) to remove free microcapsules, detached from flasks by trypsinisation and analysed. For all experiments, the cell-to-capsule ratios were 1:1, 1:5, and 1:10, respectively. All experiments were performed three times, for statistical analysis, and the standard deviations were calculated.

### 3.13. Determination of Reactive Oxygen Species (ROS)

Determination of the level of intracellular reactive oxygen species was performed using dichlorofluorescein (DCF). Cells were pre-incubated with various concentrations of nanoceria-loaded microcapsules for 24 h in a 96-well plate. After incubation, the culture medium was replaced with Hanks’ Balanced Salt Solution containing DCF (50 µM). The detection was performed using a Tecan 200 Infinity fluorescence plate reader.

### 3.14. X-Ray Irradiation

X-ray irradiation of the cells was conducted using an X-ray therapeutic machine RTM-15 (Mosrentgen, Moscow, Russia) in a dose of 15 Gy at a dose rate of 1 Gy/min, 200 kV voltage, 37.5 cm focal length and a 20 mA current. Cells were irradiated in two different experimental designs: in adherent and suspension states.

### 3.15. RT-PCR

Reverse transcription was performed with a Sileks kit (Moscow, Russia), using an oligo (dT) primer according to the manufacturer’s protocol. The produced cDNAs served as a real-time PCR matrix. For the PCR reaction, a mixture was used with SYBR Green dye (Syntol, Moscow, Russia). We used the CFX-96 amplifier (BioRad, Hercules, California, USA) or the ABI 7500 Fast Real-Time PCR System (Applied Biosystems, Foster City, California, USA). The expression of 96 genes responsible for key cell processes (Table S1) was thus determined. The analysed genes were selected from the database http://www.sabiosciences.com/for PCR profiling of different biological processes (Table S1). The level of gene transcription was normalised by the levels of expression of housekeeping genes β-actin, rplp0 (ribosomal protein, large, P0), and GAPDH (glyceraldehyde-3-phosphate dehydrogenase). Gene-specific primers were selected in the Primer Express program (Applied Biosystems, Foster City, CA, USA). Each measurement was made twice (internal repetition) and averaged for two independent samples. A sample without reverse transcription stage was used as the control. The expression data obtained were analysed using online services http://www.sabiosciences.com/, mayday-2.14 software (Centre for Bioinformatics, Tübingen, Germany) and Genesis software.

### 3.16. Statistical Data Analysis

Statistical analysis was performed using variation statistics. We determined mean values and the standard deviation of the mean. The significance of differences was determined by the Student’s *t*-test. The significance of differences in cell assays was assessed using the Mann–Whitney U criterion.

## 4. Conclusions

In this paper, we have proposed hybrid microcapsules modified with citrate-stabilised cerium oxide nanoparticles, which provide almost complete protection against X-ray irradiation. We have shown that ceria-containing microcapsules have no toxic effects and the optimal (both safe and uptake efficient) concentrations of microcapsules for human mesenchymal stem cells range from 1:10 to 1:20. After cellular uptake, nanoceria-loaded microcapsules degraded due to the action of intracellular enzymes and released nanoceria into cytoplasm. The molecular mechanisms of nanoceria radioprotective action on mesenchymal stem cells have been revealed by assessing the level of intracellular ROS, as well as by a detailed 96-genes expression analysis. Effective cellular uptake of microcapsules and uniform distribution of nanoceria increased cell viability by decreasing ROS levels, as well as modulating the genes expression involved in the development of oxidative stress, mitochondrial metabolism, apoptosis, necrosis, autophagy, inflammation etc. Hybrid ceria-containing microcapsules have been shown to provide an indirect genoprotective effect, reducing the number of cytogenetic damages in irradiated cells. These findings provide new insight into cerium oxide nanoparticles’ protective action for living beings against ionising radiation.

## Supplementary Materials

The following materials are available online: Figure S1. Survival rate of hMSC under X-ray irradiation (1–15 Gy); Figure S2. Z-stack images of hMSC after uptake of nanoceria-loaded microcapsules; Figure S3. The survival rate of hMSC after 15 Gy X-ray irradiation with different irradiation patterns and cell culture densities; Figure S4. The survival rate of hMSC after 15 Gy X-ray irradiation with different irradiation patterns and cell culture densities; Figure S5. Cell culture density of hMSC after 15 Gy X-ray irradiation with different irradiation patterns during 144 h of culturing; Figure S6. Zeta potentials of empty microcapsules and nanoceria-loaded microcapsules; Figure S7. Micrographs of hMSC after incubation (24 h) with nanoceria-loaded polyelectrolyte microcapsules (Live/Dead assay); Figure S8. High resolution SEM image of nanoceria-loaded polyelectrolyte microcapsules; Table S1. Selected gene groups for RT-PCR analysis.
