# Supplementary material for: Ceria-Containing Hybrid Multilayered Microcapsules for Enhanced Cellular Internalisation with High Radioprotection Efficiency
Source: Molecules. 2020 Jun 27;25(13):2957. doi: 10.3390/molecules25132957 (PMC7411955; doi:10.3390/molecules25132957)
Supplement: Supplementary file 1 [file molecules-25-02957-s001.pdf]

Supplementary materials

Ceria-containing Hybrid Multilayered Microcapsules for Enhanced Cellular Internalisation with High Radioprotection Efficiency

N.R. Popova[1†], A.L. Popov[1†], A.M. Ermakov[1], V.V. Reukov[2], V.K. Ivanov[3*]

[1]Institute of Theoretical and Experimental Biophysics, Russian Academy of Sciences, Pushchino, Moscow region, 142290, Russia, antonpopovleonid@gmail.com (N.R.P., A.L.P., A.M.E.)
[2]University of Georgia, 315 Dawson Hall, Athens, GA, USA (V.V.R.)
[3]Kurnakov Institute of General and Inorganic Chemistry, Russian Academy of Sciences, Moscow, 119991, Russia; van@igic.ras.ru (V.K.I.)

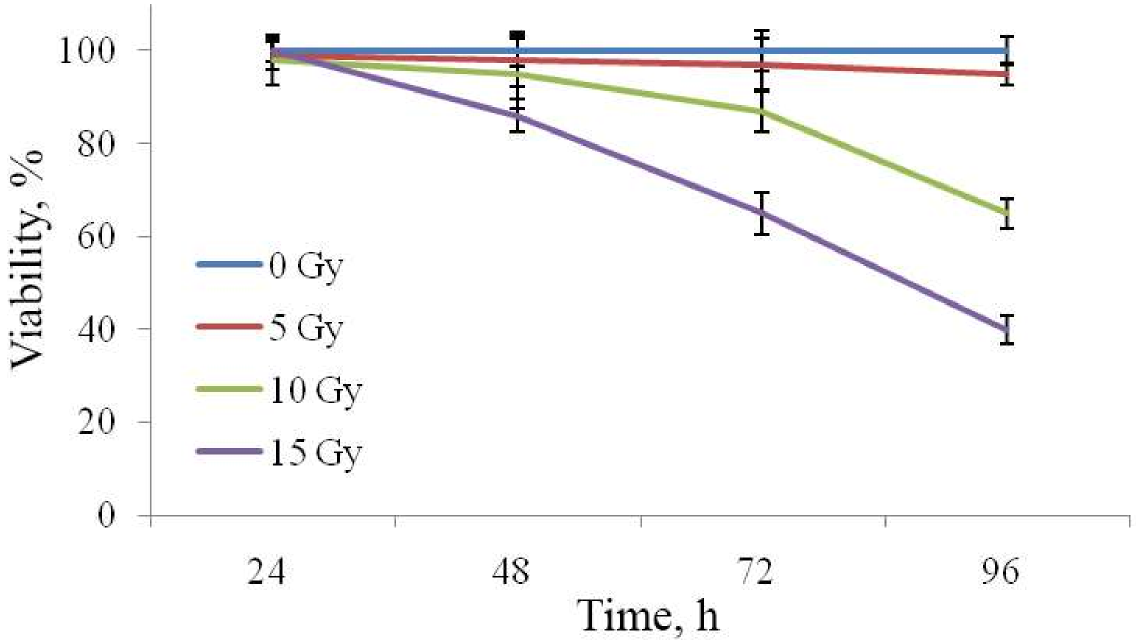

Figure S1. Survival rate of hMSC after X-ray irradiation (1-15 Gy).

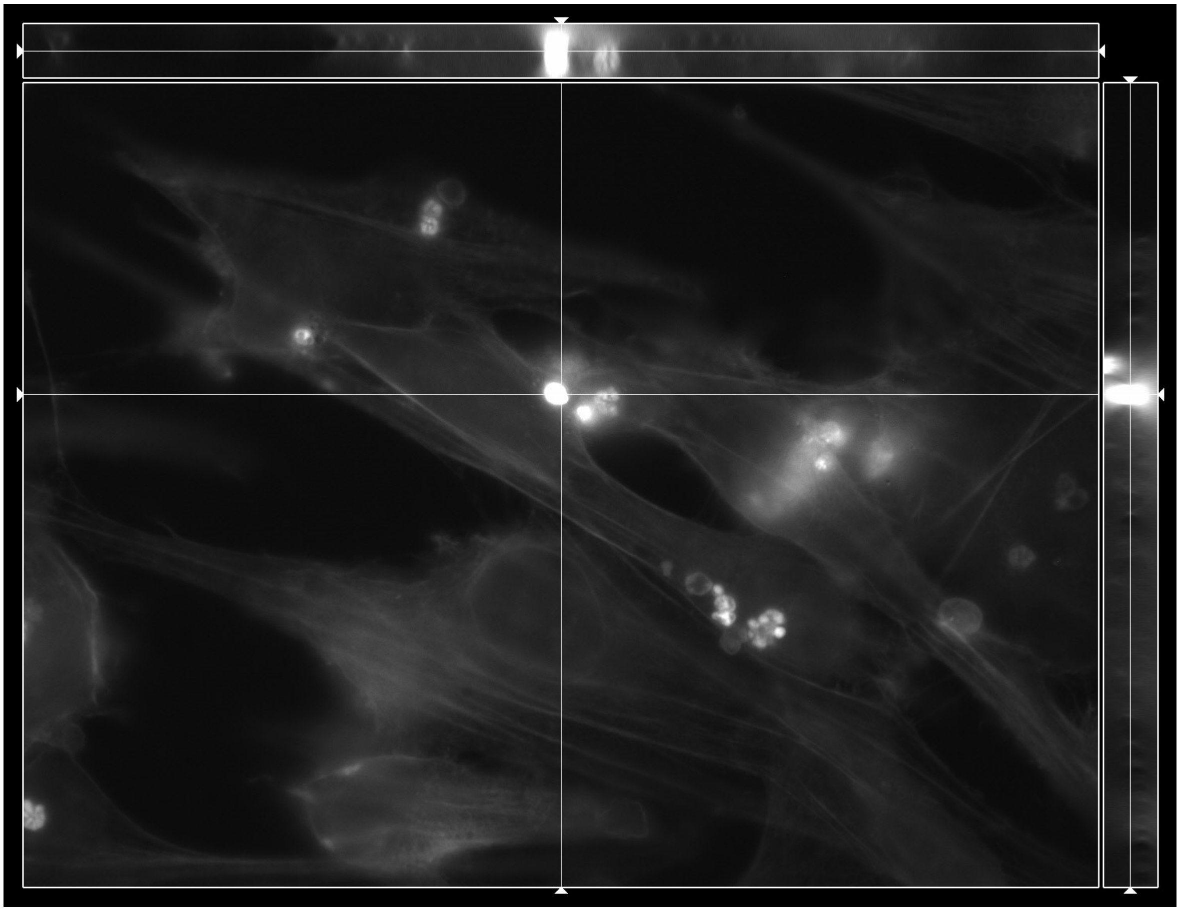

Figure S2. Z-stack images of hMSC after uptake of nanoceria-loaded microcapsules.

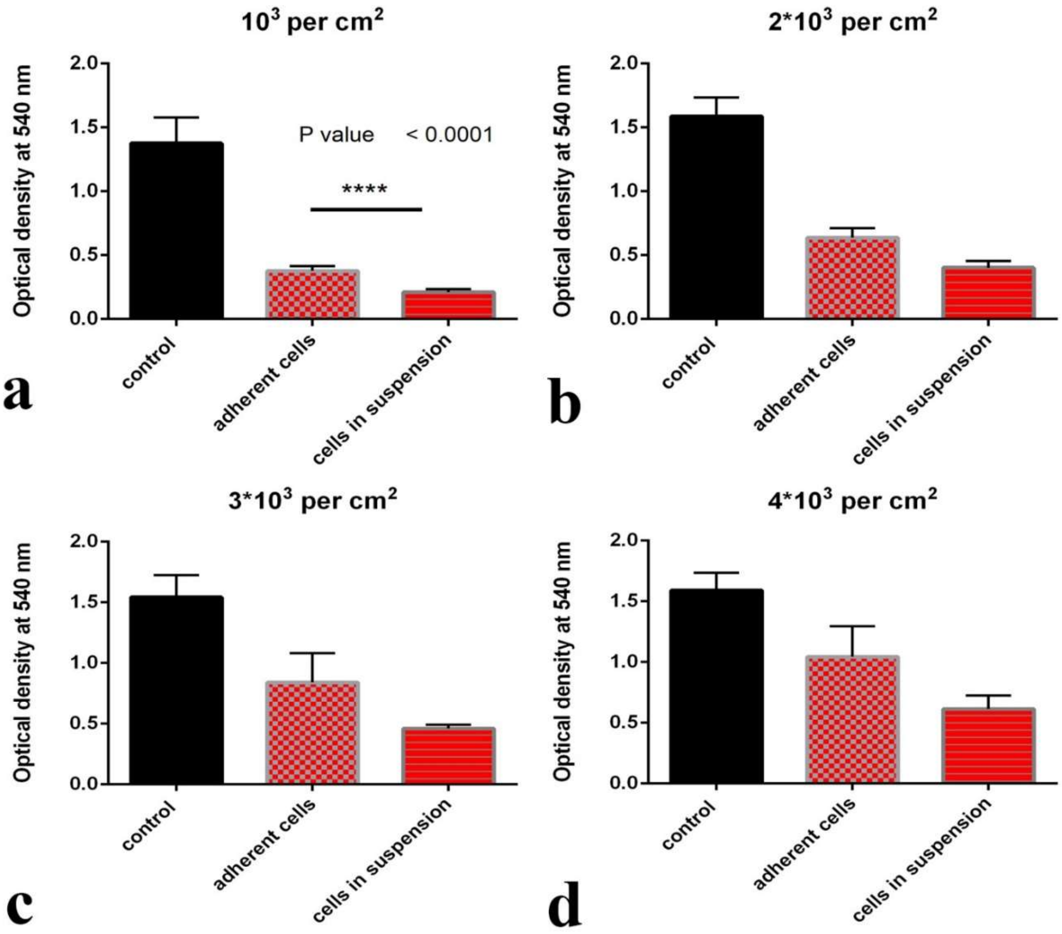

Figure S3. The survival rate of hMSC after 15 Gy X-ray irradiation with different irradiation patterns and cell culture densities.

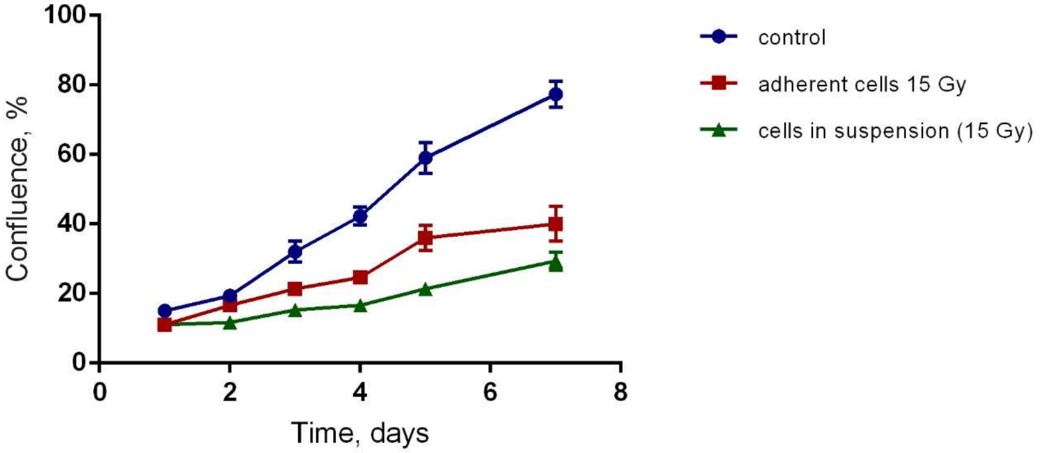

Figure S4. The survival rate of hMSC after 15 Gy X-ray irradiation with different irradiation patterns and cell culture densities.

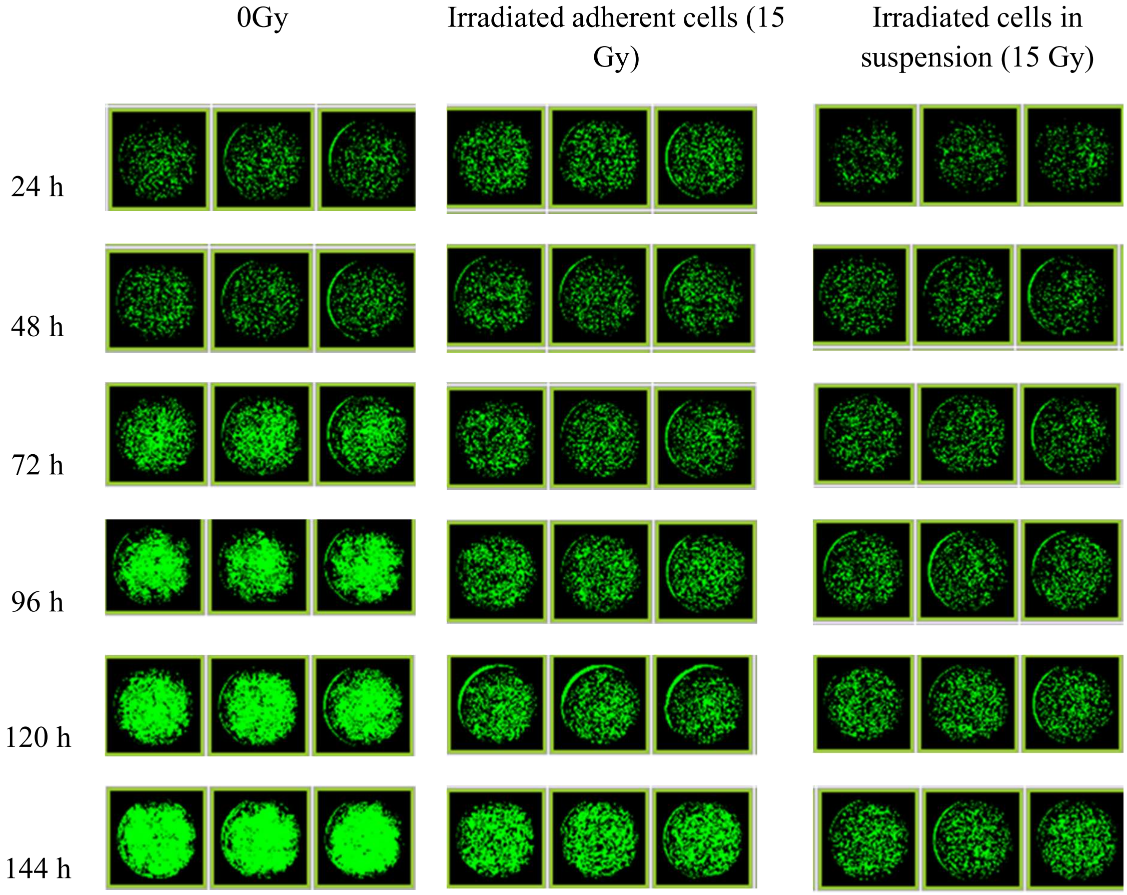

Figure S5. Cell culture density of hMSC after 15 Gy X-ray irradiation with different irradiation patterns during 144 hours of culturing.

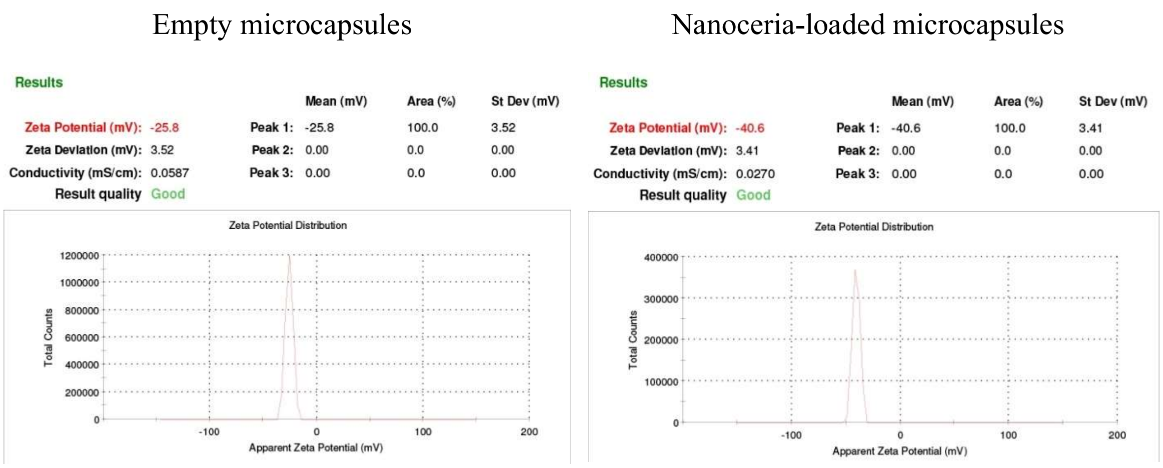

Figure S6. Zeta potentials of empty microcapsules and nanoceria-loaded microcapsules.

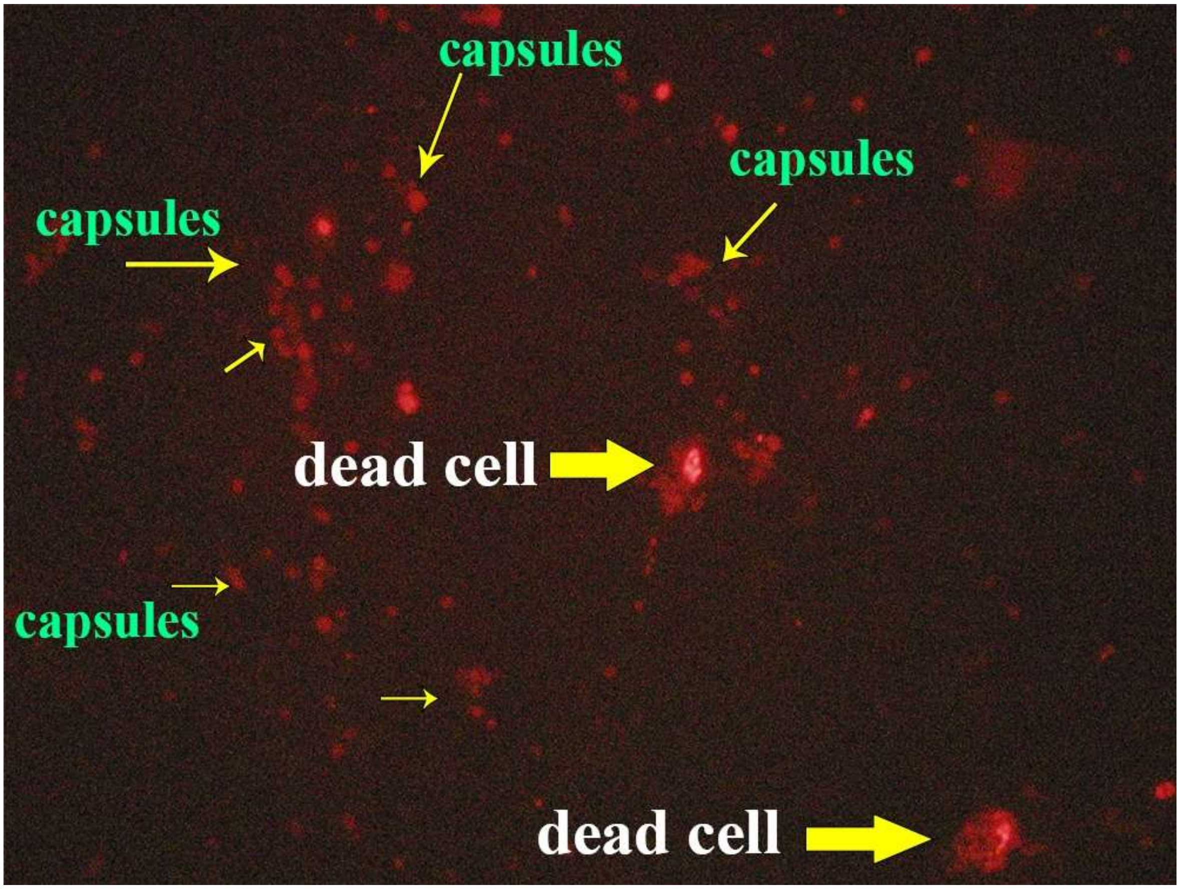

Figure S7. Micrographs of hMSC after incubation (24 h) with nanoceria-loaded polyelectrolyte microcapsules (Live/Dead assay).

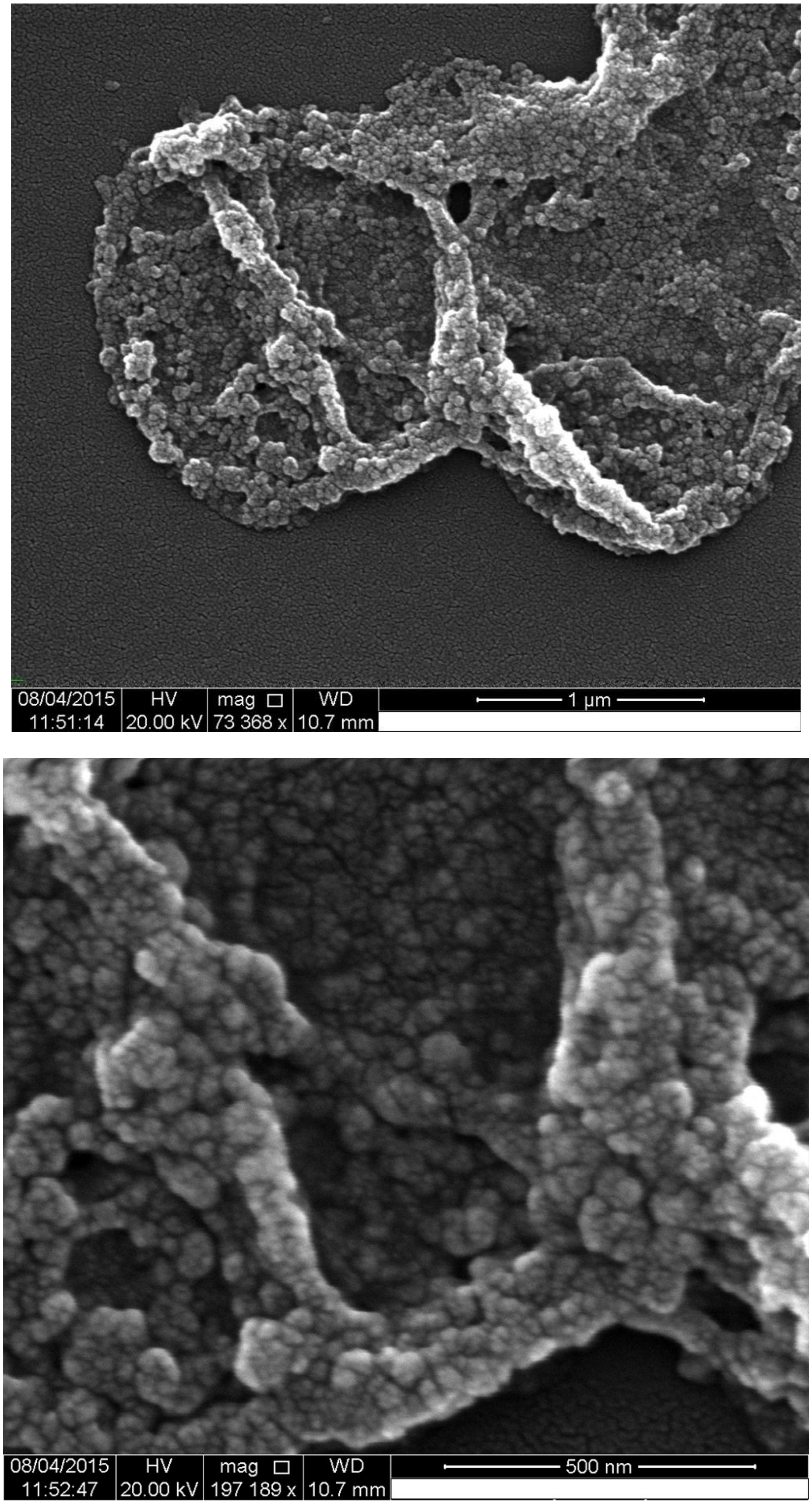

Figure S8. High resolution SEM image of nanoceria-loaded polyelectrolyte microcapsules.

**Table S1**. Selected gene groups for RT-PCR analysis.		
	Gene #	Gene Symbol
Glutathione Peroxidases (GPx)	A01	GPX1
Glutathione Peroxidases (GPx)	A02	GPX2
Glutathione Peroxidases (GPx)	A03	GPX3
Glutathione Peroxidases (GPx)	A04	GPX4
Glutathione Peroxidases (GPx)	A05	GPX5
Glutathione Peroxidases (GPx)	A06	GSTP1
Glutathione Peroxidases (GPx)	A07	GSTZ1
Peroxiredoxins (TPx)	A08	PRDX1
Peroxiredoxins (TPx)	A09	PRDX2
Peroxiredoxins (TPx)	A10	PRDX3
Peroxiredoxins (TPx)	A11	PRDX4
Peroxiredoxins (TPx)	A12	PRDX5
Peroxiredoxins (TPx)	B01	PRDX6
Other Peroxidases	B02	CAT
Other Peroxidases	B03	CYBB
Other Peroxidases	B04	CYGB
Other Peroxidases	B05	DUOX1
Other Peroxidases	B06	DUOX2
Other Peroxidases	B07	LPO
Other Peroxidases	B08	MPO
Other Peroxidases	B09	PTGS1
Other Peroxidases	B10	PTGS2
Other Antioxidants	B11	ALB
Other Antioxidants	B12	APOE

Other Antioxidants	C01	GSR
Other Antioxidants	C02	MT3
Other Antioxidants	C03	SRXN1
Other Antioxidants	C04	SOD1
Other Antioxidants	C05	SOD2
Other Antioxidants	C06	SOD3
Genes Involved in Reactive Oxygen Species (ROS) Metabolism	C07	ALOX12
Genes Involved in Reactive Oxygen Species (ROS) Metabolism	C08	NOS2
Genes Involved in Reactive Oxygen Species (ROS) Metabolism	C09	NOX4
Genes Involved in Reactive Oxygen Species (ROS) Metabolism	C10	NOX5
Genes Involved in Reactive Oxygen Species (ROS) Metabolism	C11	UCP2
Genes Involved in Reactive Oxygen Species (ROS) Metabolism	C12	AOX1
Genes Involved in Reactive Oxygen Species (ROS) Metabolism	D01	BNIP3
Genes Involved in Reactive Oxygen Species (ROS) Metabolism	D02	EPHX2
Genes Involved in Reactive Oxygen Species (ROS) Metabolism	D03	MPV17
Genes Involved in Reactive Oxygen Species (ROS) Metabolism	D04	ATOX1
Genes Involved in Reactive Oxygen Species (ROS) Metabolism	D05	CCL5
Genes Involved in Reactive Oxygen Species (ROS) Metabolism	D06	DHCR24

Genes Involved in Reactive Oxygen Species (ROS) Metabolism	D07	FOXM1
Genes Involved in Reactive Oxygen Species (ROS) Metabolism	D08	FTH1
Genes Involved in Reactive Oxygen Species (ROS) Metabolism	D09	GCLM
Genes Involved in Reactive Oxygen Species (ROS) Metabolism	D10	GSS
Genes Involved in Reactive Oxygen Species (ROS) Metabolism	D11	HMOX1
Genes Involved in Reactive Oxygen Species (ROS) Metabolism	D12	HSPA1A
Genes Involved in Reactive Oxygen Species (ROS) Metabolism	E01	MBL2
Genes Involved in Reactive Oxygen Species (ROS) Metabolism	E02	NQ01
Genes Involved in Reactive Oxygen Species (ROS) Metabolism	E03	RNF7
Genes Involved in Reactive Oxygen Species (ROS) Metabolism	E04	SIRT2
Genes Involved in Reactive Oxygen Species (ROS) Metabolism	E05	SQSTM1
Pathway Activity Signature Genes	E06	AKR1C2
Pathway Activity Signature Genes	E07	BAG2
Pathway Activity Signature Genes	E08	FHL2
Pathway Activity Signature Genes	E09	GLA
Pathway Activity Signature Genes	E10	HSP90AA1
Pathway Activity Signature Genes	E11	LHPP
Pathway Activity Signature Genes	E12	TRAPPC6A
Mitochondrial dysfunction	F01	MRPL43

Mitochondrial dysfunction	F02	NDUFB11
Mitochondrial dysfunction	F03	POLRMT
Mitochondrial dysfunction	F04	SIRT1
Mitochondrial dysfunction	F05	SIRT3
Mitochondrial dysfunction	F07	TFAM
Mitochondrial dysfunction	F08	TFB1M
Mitochondrial dysfunction	F09	TFB2M
Mitochondrial dysfunction	F10	CCS
Mitochondrial dysfunction	F11	SELENOS
Anti Apoptotic	F12	NOS2
Anti Apoptotic	G01	BCL2
Anti Apoptotic	G02	BIRC3
Anti Apoptotic	G03	MCL1
Anti Apoptotic	G04	TRAF2
Autophagy	G05	ATG3
Autophagy	G06	ATG12
Autophagy	G07	NFKB1
Autophagy	G08	RPS6KB1
Necrosis:	G09	CCDC103
Necrosis:	G10	FOXI1
Necrosis:	G11	JPH3
Necrosis:	G12	RAB25
Pro apoptotic	H01	BAX
Pro apoptotic	H02	CD40
Pro apoptotic	H03	CFLAR
Pro apoptotic	H04	FAS

Pro apoptotic	H05	TNFRSF10A
Glutathione Peroxidases (GPx)	A01	GPX1
Glutathione Peroxidases (GPx)	A02	GPX2
Glutathione Peroxidases (GPx)	A03	GPX3
Glutathione Peroxidases (GPx)	A04	GPX4
Glutathione Peroxidases (GPx)	A05	GPX5
Glutathione Peroxidases (GPx)	A06	GSTP1
Glutathione Peroxidases (GPx)	A07	GSTZ1
Peroxiredoxins (TPx)	A08	PRDX1
Peroxiredoxins (TPx)	A09	PRDX2
Peroxiredoxins (TPx)	A10	PRDX3
